# Asthma

**Published:** 1894-03-17

**Authors:** 


### ASTHMA.

Conflicting views have been advanced in explanation of the respiratory symptoms in asthma. On the one hand it is held that spasm of the bronchioles occurs, while on the other hand it has been contended that the sudden dyspnœa is due to vaso-motor phenomena, resulting in vascular turgescence. Some valuable experiments by Einthoven[1] have shown (1) that the excitation of the peripheral end of either vagus causes strong broncho-constriction, shown by increase of pressure in, and distension of the lungs; (2) that this action is abolished by nicotin or by atropin.

The author urges that bronchial spasm is capable of producing acute emphysema. Grossmann,[2] in criticising these results, reaffirms Von Basch's doctrine that engorgement of the pulmonary capillaries obstructs respiration by distending the alveoli.

Paraldehyde has been used with success in about a dozen cases of spasmodic asthma by Dr. Mackie.[3] He was led to try its effect in such cases from the fact that this remedy is a sedative largely eliminated by the breath. He found that it speedily relieved the spasm and induced sleep. Several of these cases had been already treated with all the known remedies, but every one of them pronounced the paraldehyde treatment the best they had ever tried.

He has usually given it in ℨss doses, repeated every hour or half-hour until the patient is relieved. In no case have more than three doses been required, and frequently only one was needed. In a boy aged thirteen, who had been asthmatic "from his birth," a dose of 20 minims was found to have a miraculous effect, mother and patient both declaring no drug had ever given such relief.

Aspidospermine (quebracho aspidosperma) has been clinically investigated by Bardet,[4] especially in its relations to the relief of dyspnœa. The drug increases the fulness of the respiratory movements, slows the heart, and lowers the temperature below normal. It has been found useful in the treatment of simple functional dyspnœa by Bouchard. The dose is from a quarter to half a grain up to a grain, or a solution may be given hypodermically—aspidospermine, grains iij. to water ℨiii. A small percentage of sulphuric acid may be added to maintain the drug in solution, the acid being neutralised at the moment of injection with a little bicarbonate of soda. The ordinary dose of this solution is fifteen drops hypodermically.

We have already referred to the vaso-motor theory of asthma. Kruse[5] refers to the theories relating to the pathology of bronchial asthma, but himself believes that all treatment based on altering the condition of other supposed sympathetic organs has, in the majority of cases, failed. He regards the usual cause of the malady as a morbid condition of the bronchioles themselves. This author extols the benefits derived from the inhalation of sea air, a therapeutic factor on which at present insufficient stress is laid. He has himself watched a large number of cases during their temporary residence by the sea, and was impressed by the general improvement which followed. In fifty-six cases an after-history was obtained, and among these patients twenty-nine were apparently permanently cured, and in sixteen other cases complicated with emphysema the improvement maintained itself for several months. The author considers that the greater density of sea air, the higher degree of moisture, and the absence of dust, &c., assisted in the above results.

[1] Pflüger's Arch., 1892; Brain, parts I. and II., 1893. [2] Pflüger's Arch., vol. LII., p. 415; Brain, loc. cit. [3] Brit. Med. Journ., Jan. 14, 1893. [4] Rev. Int. de Bibl. Méd., Apr. 10, 1893. [5] Wien. Med., Woch., Nos. 22 and 23, 1893; Brit. Med. Journ., July 29.

